# Polyhedral Oligomeric Sesquioxane Cross-Linked Chitosan-Based Multi-Effective Aerogel Preparation and Its Water-Driven Recovery Mechanism

**DOI:** 10.3390/gels10040279

**Published:** 2024-04-20

**Authors:** Yang Liu, Mingjian Ma, Yuan Shen, Zhengdong Zhao, Xuefei Wang, Jiaqi Wang, Jiangbo Pan, Di Wang, Chengyu Wang, Jian Li

**Affiliations:** 1 Key Laboratory of Bio-Based Material Science and Technology, Ministry of Education, Northeast Forestry University, Harbin 150040, China; hljqaliuyang@163.com (Y.L.); 18847042762@163.com (M.M.); jlaushawn@yeah.net (Y.S.); zhaozd6866@163.com (Z.Z.); fei1807352093@163.com (X.W.); w1531565456@163.com (J.W.); pjb0725@163.com (J.P.); wangcy@nefu.edu.cn (C.W.); nefujianli@163.com (J.L.); 2 College of Material Science and Engineering, Northeast Forestry University, Harbin 150040, China

**Keywords:** chitosan-based aerogel, EP-POSS, water-driven recovery, water purification

## Abstract

The use of environmentally friendly and non-toxic biomass-based interfacial solar water evaporators has been widely reported as a method for water purification in recent years. However, the poor stability of the water transport layer made from biomass materials and its susceptibility to deformation when exposed to harsh environments limit its practical application. To address this issue, water-driven recovery aerogel (PCS) was prepared by cross-linking epoxy-based polyhedral oligomeric silsesquioxane (EP-POSS) epoxy groups with chitosan (CS) amino groups. The results demonstrate that PCS exhibits excellent water-driven recovery performance, regaining its original volume within a very short time (1.9 s) after strong compression (ε > 80%). Moreover, PCS has a water absorption rate of 2.67 mm s^−1^ and exhibits an excellent water absorption capacity of 22.09 g g^−1^ even after ten cycles of absorption-removal. Furthermore, a photothermal evaporator (PCH) was prepared by loading the top layer with hydrothermally reacted tannins (HAs) and Zn^2+^ complexes. The results indicate that PCH achieves an impressive evaporation rate of 1.89 kg m^−2^ h^−1^ under one sun illumination. Additionally, due to the antimicrobial properties of Zn^2+^, PCH shows inhibitory effects against Staphylococcus aureus and Escherichia coli, thereby extending the application of solar water evaporators to include antimicrobial purification in natural waters.

## 1. Introduction

In recent years, the scarcity of freshwater resources has emerged as a global problem that cannot be ignored [1]. Conventional water purification techniques like atmospheric water harvesting [2] and seawater desalination [3] not only consume substantial energy but also inflict significant damage on the surrounding environment [4]. In comparison to traditional solar evaporation technology, interfacial solar evaporation technology constructs a Janus structure comprising a photothermic conversion layer and a water transport layer using suitable materials [5]. That can improve the evaporation rate and solar energy conversion efficiency by enhancing the light absorption capacity, reducing the thermal conductivity, and optimizing the transport of the water body and so on [6,7,8], which has become the most widely used new solar evaporation technology at present [9].

The primary function of the water transport layer is to timely convey bulk water to the evaporator surface while minimizing heat loss and augmenting surface warming effects. Consequently, it significantly influences the evaporation rate of interfacial solar evaporators. Currently, three-dimensional porous structures such as foam [10] and aerogel [11] are predominantly employed for constructing the water transport layer. Renewable biomass materials are extensively utilized in fabricating the water transport layer due to their environmentally friendly nature. As one of the most abundant biomass materials, chitosan (CS) is rich in hydroxyl and amino groups, which can provide a large number of reaction sites [12], and is widely used in the preparation of biomass aerogels. CS-based materials are currently prevalent in various fields including wastewater treatment [13], antimicrobial activities [14,15], food preservation [16], sound absorption [17], reinforcement filler [18,19], drug delivery [20], oil spill cleanup [21,22], healing wounds [23,24], etc. However, due to the poor mechanical properties and unstable structure of biomass aerogels [25], CS-based aerogels have disadvantages. Therefore, in practical applications, the water transport layer made of CS materials are susceptible to external interference, such as extrusion from external forces leading to evaporator deformation and a decreased water transport rate, which affects the evaporation rate. At present, only a few water-driven recovery aerogels have been developed using CS as a raw material due to the aforementioned shortcomings of CS aerogels. Therefore, it is necessary to modify or cross-link CS materials with other components to improve their limitations.

Currently, CS as a base aerogel is usually cross-linked and modified with glutaraldehyde [26], epichlorohydrin [27], and other organic cross-linking agents [28], as they react with the amino groups of the CS to improve the stability and mechanical properties of the aerogel. However, most organic cross-linking agents cause significant environmental pollution; therefore, their application in solar water evaporators may lead to secondary pollution concerns. Therefore, known for its excellent biocompatibility and non-toxicity [29], polyhedral oligomeric sesquioxane (POSS) is a promising choice. POSS is considered the smallest silica particle available [30] that can effectively reduce stress concentration within matrix materials through its unique nano-size effect while absorbing energy and providing good mechanical properties. POSS material combines the reactive properties of organic materials with the excellent physical properties of inorganic materials at a molecular level [31], which can enhance the various aspects of the materials such as heat resistance [32], flame retardancy [33], and mechanical properties [34]. In addition, the water-driven recovery of the aerogel can be achieved by the formation of hydrogen bonds through the combination of water with Si-OH in the irregular POSS. By incorporating POSS into CS-based materials, we can overcome the limitations associated with CS-based materials by leveraging the unique properties offered by POSS and endow CS-based aerogels with water-driven recovery capabilities. Through the function of water-driven recovery, it realizes the rapid recovery of the CS water transport layer after receiving the extrusion of external force, and solves the problem of the practical use of the CS water transport layer in harsh environments.

The main function of the photothermal layer, as another important component of the solar evaporator, is to absorb solar energy and convert it into the heat energy required for water evaporation. Compared to other photothermal materials, inexpensive, simple to prepare, and photothermal-convertible tannic acid–metal complexes offer a favorable option. Tannin, a natural plant polyphenol widely found in plant barks, roots, and leaves [35] contains numerous phenolic hydroxyl groups [36]. These groups enable tannin to form coordination reactions with metal ions [37], resulting in tannic metal complexes that exhibit excellent photothermic properties by absorbing sunlight energy through the conjugated structure of the tannin and transferring it to metal ions. This promotes electronic resonance and converts optical energy into heat energy [38]. And, the simple hydrothermal treatment of tannins (HAs) can increase its light absorption and improve its energy absorption capacity. However, the presence of metal ions in water pollution cannot be ignored; therefore, according to guidelines from the World Health Organization and drinking water standards issued by countries worldwide [39], Zn^2+^ has a maximum allowable concentration far exceeding that of Fe^3+^, making it suitable for coordination with tannin to generate metal complexes for efficient photothermic conversion. Additionally, the Zn^2+^ demonstrates potent inhibitory effects against both Escherichia coli and Staphylococcus aureus, making it a popular and low-cost antimicrobial agent. The use of HA-Zn^2+^ complexes as photothermic materials enable solar evaporators to perform various functions, such as antimicrobial activity and water evaporation in natural waters.

The present study aims to enhance the mechanical properties and stability of CS-based solar water evaporators for improved adaptability in harsh environments. To achieve this, triethoxy (3-epoxypropyl oxypropyl) silane was synthesized into epoxy-based polyhedral oligomeric silsesquioxane (EP-POSS), which was then cross-linked with CS as a cross-linking agent and freeze-dried to obtain aerogel (PCS). Notably, our PCS demonstrated remarkable self-recovery ability driven by water, along with superior mechanical strength and water absorption capacity compared to pure CS aerogels. Furthermore, interfacial photothermic aerogels (PCHs) were developed by incorporating HA-Zn^2+^ complexes onto the top of the PCH for efficient solar-driven evaporation. And PCH exhibited exceptional performance in sewage purification and antimicrobial activities. Consequently, PCH holds great potential for applications in water purification under challenging environmental conditions.

## 2. Results and Discussion

### 2.1. PCS Morphology and Structure

**Figure 1:**
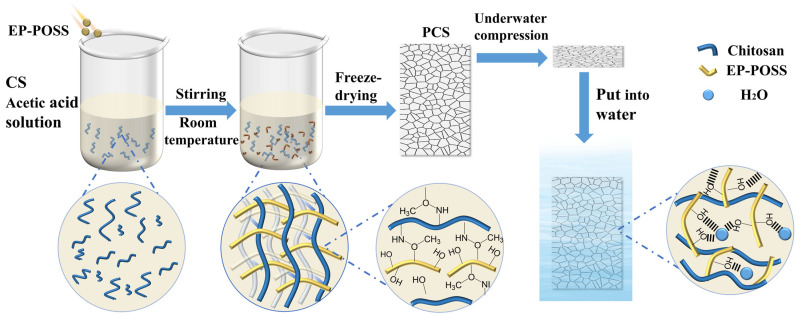
The preparation of the PCS by freeze-drying after the cross-linking of the amino group and epoxide group and the mechanism of the water-driven self-recovery were studied.

In this study, EP-POSS was synthesized in a single step through silane hydrolysis, as shown in Figure 1, and organic–inorganic hybrid materials were successfully introduced into biomass macromolecular chains by cross-linking the epoxy group of the EP-POSS with the amino group of the CS under acidic conditions. The resulting PCS was then prepared by freeze-drying, forming a PCS gel with a three-dimensional network pore structure. This structure was achieved through the direct sublimation of frozen ice crystals during freeze-drying, resulting in an ultra-lightweight aerogel with a density of only 11.33 mg cm^−3^. And, the method allows for the simple and convenient production of aerogels of various sizes by utilizing molds of different sizes. Additionally, the volumetric shrinkage of the aerogel after freeze-drying is negligible. On this basis, we prepared PCHs by loading HA-Zn^2+^ complexes onto the top of the PCS.

**Figure 2:**
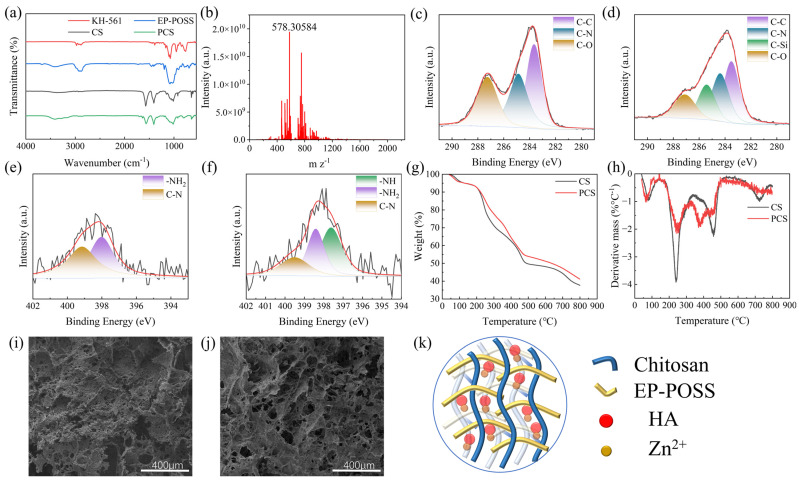
(**a**) FTIR spectra of CS, KH-561, EP-POSS, and PCS. (**b**) FT-ICR-MS spectra of EP-POSS. XPS spectra. (**c**) Pure CS aerogel C1s spectra. (**d**) PCS C1s spectra. (**e**) Pure CS aerogel N1s spectra. (**f**) PCS N1s spectra. (**g**) TG chart of pure CS aerogel and PCS. (**h**) DTG chart of pure CS aerogel and PCS. SEM images. (**i**) Pure CS aerogel. (**j**) PCS. (**k**) Schematic internal structure of PCH.

The FT-IR spectra of the POSS and PCS are shown in Figure 2a. The broad peak of the EP-POSS at 3100–3600 cm^−1^ is a stretching vibrational absorption peak of ν(O-H), which indicates that the hydrolysis of silanes occurs in the presence of water. The stretching vibrational absorption peak of ν(Si-O-Si) at 1083 cm^−1^ suggests that the condensation of Si-OH occurs in the presence of the acid as a catalyst so as to allow the Si-O-Si structure to be generated, and the presence of a hydroxyl peak indicated that the reaction is not carried out completely. The characteristic absorption peak of the epoxy group at 911 cm^−1^ indicated that the epoxy group does not undergo ring opening under the action of the acid. In order to further prove the molecular structure of the EP-POSS, it was subjected to FT-ICR-MS, and the mass spectral results are shown in Figure 2b. The peak located at 1337.55068 is a complete hexahedral cage of the EP-POSS, whose content is very small, and the peak located at 578.30584 is mainly attributed to [C_20_H_42_Si_3_O_13_ + H]^+^, as shown in Figure S1. Combining the mass spectrometry and the infrared analysis results, it can be seen that most of the EP-POSS prepared directly by silane hydrolysis are silane chains that do not form a complete cage structure, and only a small portion of them is formed. The broad peak of the PCS located at 3050–3600 cm^−1^ is the stretching vibrational absorption peak of the ν(O-H), which indicates the existence of the strong intermolecular and intramolecular hydroxyl group with hydrogen bonding. The stretching vibrational absorption peaks for ν(-CH_2_) and ν(-CH_3_) at 2866 cm^−1^ to 2937 cm^−1^, the distorted vibrational absorption peaks for δ(-NH_2_) at 1573 cm^−1^, and the distorted vibrational absorption peaks for δ(-OH) at 1259 cm^−1^. The stretching vibrational absorption peaks for ν(Si-O) at 800 cm^−1^, the δ(-NH_2_) characteristic absorption peak at 1573 cm^−1^ is weakened and shifted to a lower wave number to 1563 cm^−1^, the δ(N-H) absorption peak [40] at 1412 cm^−1^ is shifted to 1406 cm^−1^, and the ν(C-O) absorption band on the chitosan skeleton at 1000–1200 cm^−1^, with the internal absorption peaks shifted to different degrees. These suggest that the amino group of CS reacts with the EP-POSS epoxy group to cross-link to form an aerogel.

To further demonstrate the cross-linking of the EP-POSS with CS, the aerogel is subjected to XPS test, and the binding energy peaks of the aerogel are shown in Figure 2c–f. The binding energy peaks of the pure CS aerogel at 283.87 eV, 285.46 eV, and 287.27 eV in the C1s binding energy spectrum denote C-C, C-N, and C-O, respectively, while the binding energy peaks at 398.02 eV and 399.15 eV in the N1s binding energy spectrum denote is -NH_2_ and C-N, respectively. After the addition of the EP-POSS, there is a new peak located at 285.4 eV in the C1s binding energy spectrum of the PCS, which is attributed to C-Si. Also, a new peak located at 397.62 eV in the N1s spectrum is ascribed to -NH-. Moreover, the peak of the -NH_2_ at 398.02 eV and C-N at 399.15 eV in the binding energy spectrum of N1s after the addition of the EP-POSS are shifted to the higher binding energy at 398.41 eV, 399.5 eV, indicating that the amino group of the CS cross-links with the epoxide group of the EP-POSS and the primary amine changes to secondary amine. To sum up, it can be proved that the EP-POSS has been cross-linked with the CS through IR spectra and XPS photoelectron spectroscopy.

The thermal decomposition behavior of the PCS is shown in Figure 2g,h. It is mainly divided into three stages. The first stage is at 40–100 °C and 100–200 °C, which is mainly the evaporation of the physically adsorbed free water and internally bound water [41]. The second stage is at 200–300 °C, which contains the decomposition of the CS sugar chains and organic groups on the side face of the EP-POSS [42]. The third stage at 300–800 °C mainly consisted of the complete carbonylation decomposition of the CS [43]. The carbon residual content of the aerogel increases from 37.6% to 41.2% after the addition of the EP-POSS, and the temperature at which the maximum thermal decomposition rate is reached increases from 239.5 °C to 250.2 °C, with a decrease in the maximum thermal decomposition rate, which indicates that the thermal stability of the CS-based aerogel is significantly improved after the addition of the EP-POSS.

According to the SEM of the PCS shown in Figure 2i,j, it can be seen that the pure CS aerogel without the EP-POSS is made up of the CS fragments of different sizes stacked on top of each other, which has few pore structures and is poorly connected to each other. This is due to pure CS aerogel being physically cross-linked by the ionic bonding cross-linking method, which makes the pure CS aerogel highly susceptible to decomposition in a liquid environment [25]. After the addition of the EP-POSS, the pores of the aerogel become more abundant, forming a honeycomb-like three-dimensional pore structure ranging from tens of micrometers to several hundred micrometers. This is due to the fact that the reaction between the epoxy group of the EP-POSS and the amino group of the CS makes it a much tighter chemical cross-linking, which makes its structure more stable. The rich pore structure can form a stronger capillary force when transporting water, which is more favorable for the transport of water [44]. According to the BET test, the average adsorption pore size of the PCS is about 54.45 nm (Figure S4).

### 2.2. Water-Driven Recovery Properties and Mechanism of PCS

**Figure 3:**
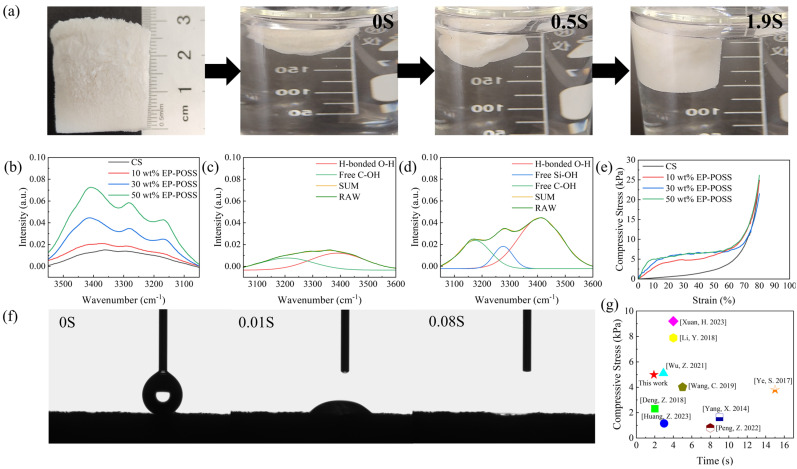
(**a**) Image of the PCS-compressed water-induced recovery process. (**b**) FTIR spectra of the hydroxyl absorbance of the PCS with different ratios of the EP-POSS added. Gaussian fitting. (**c**) Fitted spectra of the pure CS aerogel. (**d**) Fitted spectra of the PCS. (**e**) Compressive stress–strain curves of the pure CS aerogel with the addition of different ratios of the EP-POSS. (**f**) Contact angle image of the PCS aerogel. (**g**) Comparison of the mechanical properties and water-driven recovery of the aerogels in this study with previous [Ye, S. 2017] [45], [Huang, Z. 2023] [49], [Deng, Z. 2018] [50], [Xuan, H. 2023] [51], [Yang, X. 2014] [52], [Peng, Z. 2022] [53], [Li, Y. 2018] [54], [Wang, C. 2019] [55], [Wu, Z. 2021] [56].

The water contact angle of the PCS is shown in Figure 3f. A contact angle of 52.4° between the PCS and the aerogel is formed at the moment of the droplet’s fall, which is smaller than the 86.5° (Figure S6) of the pure CS aerogel, and the PCS is completely absorbed the water within 0.08 s (Video S1), whereas the pure CS aerogel requires 0.2 s (Video S2), which shows that the PCS has excellent hydrophilicity and strong water absorption capacity. The water-driven recovery of the PCS is excellent, as shown in Figure 3a. The PCS with a height of 25 mm was immersed in the water and then strongly squeezed (ε > 80%) to a height of 5 mm PCS sheet. Then, when the pressure is released, air cannot occupy the pores of the PCS and the aerogel cannot return to its initial volume. However, after re-inserting the PCS into the water, the PCS sheet recovers 50% of its volume within 1 s and almost completely recovers its initial shape within 1.9 s (Video S3). This indicates that water enters and occupies the pores of the compressed aerogel; hence, the aerogel expands to its original volume and shape. This rapid water-driven recovery property is related to the cross-linking of the chain EP-POSS with the introduction of the hydroxyl groups [45].

To further demonstrate the relationship between this water-driven self-recovery phenomenon and the PCS hydroxyl groups, the infrared absorption spectra of the PCS and CS aerogels were analyzed by peak splitting. Figure 3b shows the FT-IR absorbance profiles of the PCS with different ratios of the EP-POSS added, from which it can be seen that the ν(O-H) peak changes significantly, from a broad peak containing two absorption peaks to a broad peak containing three absorption peaks, and the intensity of the ν(O-H) peak increases significantly with the addition of the EP-POSS. The FT-IR spectra of the CS aerogel and PCS are obtained through Gaussian fitting analysis, as shown in Figure 3c,d. After fitting the ν(O-H) peaks, the absorption peaks are formed by hydrogen-bonded hydroxyls located at 3390–3415 cm^−1^, and free carbon hydroxyls located at 3174–3209 cm^−1^, which can be seen in the bifurcation diagram of the pure CS aerogel. After adding the EP-POSS for cross-linking, the ν(O-H) peaks change significantly, and there appear absorption peaks at 3260–3285 cm^−1^ formed by free Si-OH after fitting the peaks [46]. The spectra show that the incompletely condensed chain EP-POSS introduces a large amount of Si-OH into the aerogel after the introduction of the chain EP-POSS cross-linking, and the formation of the hydrogen bonding between the Si-OH and CS makes the PCS structure more stable [47]. However, the addition of water causes the hydrogen bonding between Si-OH and CS to be broken after immersing the PCS in water, and the pores of the aerogel are occupied by water and the PCS became soft by the water wetting. After compressing the PCS, the residual moisture formed hydrogen bonds with the hydroxyl groups in the aerogel, and air could not disrupt this structure to occupy the pores and restore it to its original shape. When the PCS is put into the water again, due to its strong water absorption, enough water re-enters the PCS and forms hydrogen bonds with the hydroxyl groups in the PCS [48], which rapidly fills up the pore structure of the PCS and completely restores the original shape. Therefore, it can be proved that the hydroxyl group, which is introduced through the addition of the EP-POSS, combines with water to form hydrogen bonds, thereby causing the PCS to exhibit water-driven recovery behavior. It is the introduction of hydroxyl groups and the EP-POSS that allows the PCS to exhibit superior mechanical properties and recovery compared to previous water-driven recovery aerogels.

### 2.3. Mechanical Properties of PCS

The compressive stress–strain curves of the PCS and pure CS aerogel are shown in Figure 3e. At up to 80% strain, it can be found that the compressive stress–strain curves can be divided into three regions, which are the elastic region (0 < ε < 10%) where the PCS undergoes elastic deformation, the yield region (10% < ε < 60%) where the pore structure of the PCS changes continuously with increasing pressure and undergoes irreversible plastic deformation, and a dense region (ε < 60%) where the PCS structure undergoes complete collapse failure under strong pressure. At low stresses, the 10% strain corresponds to the stresses of 2.77 kPa, 3.95 kPa, and 4.99 kPa, and the Young’s modulus of 27.39 kPa, 42.42 kPa, and 56.52 kPa, which are much higher than those of the pure CS aerogel of 0.25 kPa and 2.26 kPa, respectively, when the proportion of the EP-POSS is 10 wt%, 30 wt%, and 50 wt%. The results show that the compressive strength of the PCS increases with the increase in the EP-POSS content. The main reason is that when the content of the EP-POSS increases, the degree of cross-linking of the PCS is enhanced, the hydrogen bonds formed between the Si-OH and CS are increased, and the structure of the PCS is more stable so that the aerogels’ mechanical properties are improved [47]. The improved mechanical properties may also be related to the unique nano-size effect of the EP-POSS, which may positively affect the mechanical properties of the PCS [57].

### 2.4. Water Transport Properties of PCS

**Figure 4:**
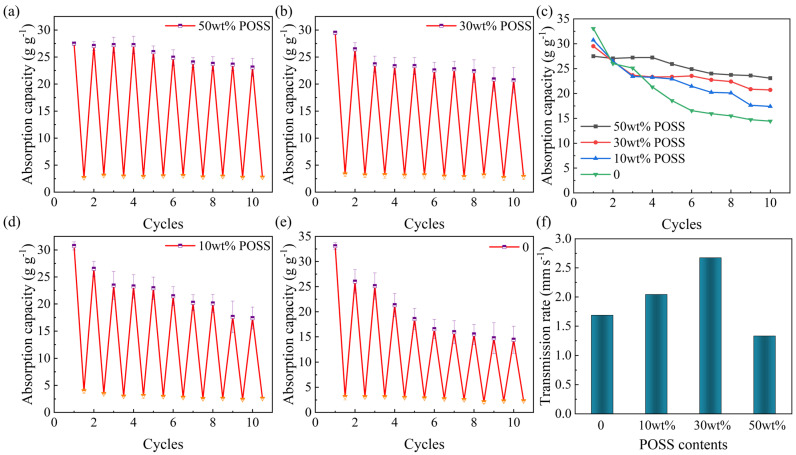
Compression–expansion cycle water absorption test of the pure CS aerogel and different ratios of PCS (**a**) 50 wt% POSS, (**b**) 30 wt% POSS, (**d**) 10 wt% POSS, and (**e**)pure CS aerogel. (**c**) Comparison of the compression–expansion cycle water absorption with different ratios of PCS. (**f**) The water transport rate of the pure CS aerogel and different ratios of PCS.

In order to investigate the cyclic usability of the PCS compression–expansion and changes in water absorption capacity, the PCS went through the water absorption expansion–dehydration compression cycle 10 times. The results are shown in Figure 4a–e. Pure CS aerogel has a maximum water absorption of 33.07 g g^−1^ when it first enters water; however, it produces hydrogen bonds in the presence of water, destroying the cross-linking of the aerogel [58], at which point the aerogel becomes transparent (Video S4). The pure CS aerogel broke after compression and drainage (Video S5), and the water absorption dropped sharply. The second time it is only 26.01 g g^−1^, less than 80% of the first water absorption, and the tenth time it is only 14.44 g g^−1^, less than 50% of the first water absorption. With the increase in the EP-POSS incorporation content, the amount of water absorbed by PCS after compression–absorption was gradually stabilized. The maximum water absorption capacity of the PCS containing 50 wt% EP-POSS in the first suction (Video S6) is 27.50 g g^−1^ after compression and drainage, and the second water-absorbing capacity is 27.06 g g^−1^, reaching 98.4% of the first time. The water-absorbing capacity after ten compression and drainage cycles is still 23.09 g g^−1^, reaching 83.9% of the first time, and there is no obvious breakage of the PCS in the whole process of suction, compression, and drainage cycles. It shows that it can still have good water transport performance and keep the evaporation continuity of the photothermal layer after several times of severe external extrusions.

Figure 4f shows the water transport rate of the aerogel, and the aerogel end is vertically contacted to the water surface to record the time required for the water to rise to 20 mm. It can be seen that the water transport rate of the pure CS aerogel is 1.69 mm s^−1^, while that of the PCS with 30 wt% POSS reaches 2.67 mm s^−1^, which is higher than the other reported water transport layer [59,60]. The enhanced water transport rate may be due to the introduction of hydrophilic hydroxyl groups in the chain EP-POSS cross-linked with CS. However, the water transport rate of the PCS decreased to 1.33 mm s^−1^ when the content of the EP-POSS was increased to 50 wt%, which could be attributed to the introduction of the excessive Si-O-Si structure into the PCS. The Si-O-Si structure acts as a hydrophobic group [61], and the introduction of a small amount may lead to a faster upward escape of water molecules, while the introduction of too much may have a greater negative effect. To demonstrate that the PCS can be applied to a conditioned harsh environment without breakage, it was further placed in an ultrasonic cleaner with a power of 360 W (Video S7, Figure S7) to simulate the external environment. There was no significant change in the aerogel after ultrasonic vibrating for 30 min, indicating that the aerogel can be applied to be used in harsh environments.

### 2.5. Photothermal Evaporation Properties of PCH

**Figure 5:**
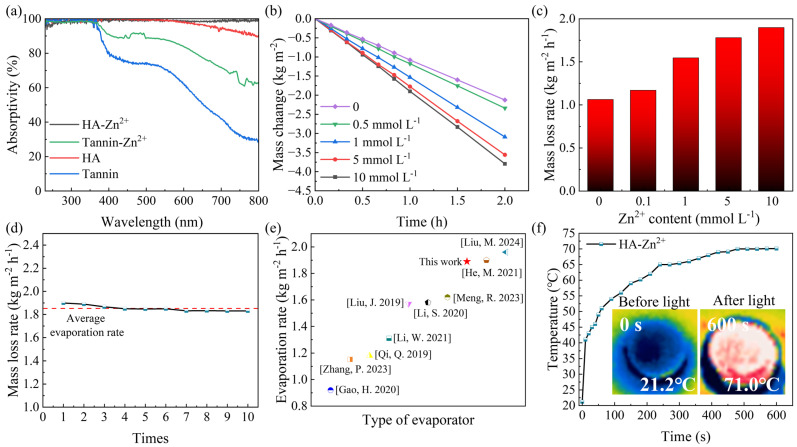
(**a**) UV-Vis spectra of different photothermal layers in wet state. (**b**) Loss of water mass in the light intensity of one sun. (**c**) The rate of evaporation in the light intensity of one sun. (**d**) The evaporation rate of 10 consecutive evaporations. (**e**) Comparison of evaporation rates between this work and other literature under the same solar irradiation condition [Gao, H. 2020] [62], [Zhang, P. 2023] [63], [He, M. 2021] [64], [Li, S. 2020] [65], [Liu, J. 2019] [66], [Liu, M. 2024] [67], [Meng, R. 2023] [68], [Qi, Q. 2019] [69], [Li, W. 2021] [70]. (**f**) Infrared thermal imaging and temperature change curve of the dried PCH under one solar illumination intensity.

The photothermal effect of aerogels is realized through the coordination of HA with metal ions. The UV-Vis spectra of the aerogels loaded with different photothermal layers are shown in Figure 5a. In the visible band, the average absorptivity of the HA-Zn^2+^ complex aerogel can reach 98.9%, which is higher than that of the aerogel with HA alone and the aerogel with tannin alone. Regarding the absorbance versus the color of the aerogels, the higher the absorbance in the visible band, the darker the color. In contrast, the average absorption rate of the photothermal layer loaded with tannin-Zn^2+^ is only 81.9%, indicating that HA-Zn^2+^ can absorb more energy than tannin-Zn^2+^.

The relationship between photothermal performance and absorbance is verified by photothermal evaporation experiments. Under the irradiation of a full-band xenon lamp simulating sunlight, Figure 5b,c show the HA-Zn^2+^ evaporator loaded with different concentrations of Zn^2+^ ion solutions undergoing continuous photothermal evaporation tests for 2 h. As the Zn^2+^ ion concentration increased, the evaporation rate of the PCH rose by 78.6%, reaching a rate of 1.89 kg m^−2^ h^−1^. The evaporation rate is higher than most evaporators that use biomass material as a substrate (Figure 5e). However, when the concentration of the Zn^2+^ ion solution is increased from 5 mmol L^−1^ to 10 mmol L^−1^, the increase in the evaporation rate is not significant. This may be due to the upper limit of the coordination capacity of the HA. In order to demonstrate the continuous availability of the PCH, 10 consecutive photothermal evaporation tests were performed on the PCH loaded with the highest complexing concentration, and the results are shown in Figure 5d. Benefiting from the excellent underwater stability and water transport of the PCS, the average evaporation rate of the PCH is 1.85 kg m^−2^ h^−1^ in 10 consecutive evaporation experiments, which is stabilized at 96.4% of the maximum evaporation rate after 7 evaporation experiments. The experimental results show that the PCH aerogel has the potential for long-term use.

The infrared thermal imaging of the PCH under simulated sunlight is shown in Figure 5f. Under the light intensity of one sun, the surface temperature of the PCH can rapidly increase to 51.0 °C in one minute and 65.4 °C in five minutes, and the temperature reaches a steady maximum of 70.1 °C in ten minutes. The maximum temperature of the PCS not loaded with a photothermal layer is only 31.1 °C after 10 min of irradiation. And, the maximum temperature of the PCS loaded with HA is only 59.7 °C, while the maximum temperature of the PCS loaded with tannin-Zn^2+^ is only 48.3 °C after 10 min of irradiation (Figure S9). The results indicate that the energy absorbed by the complex can be released as a part of the thermal energy through the vibration of the metal ions by the coordination of the TA with metal ions, thus enhancing its photothermal conversion ability [38].

### 2.6. Photothermal Purification Performance

**Figure 6:**
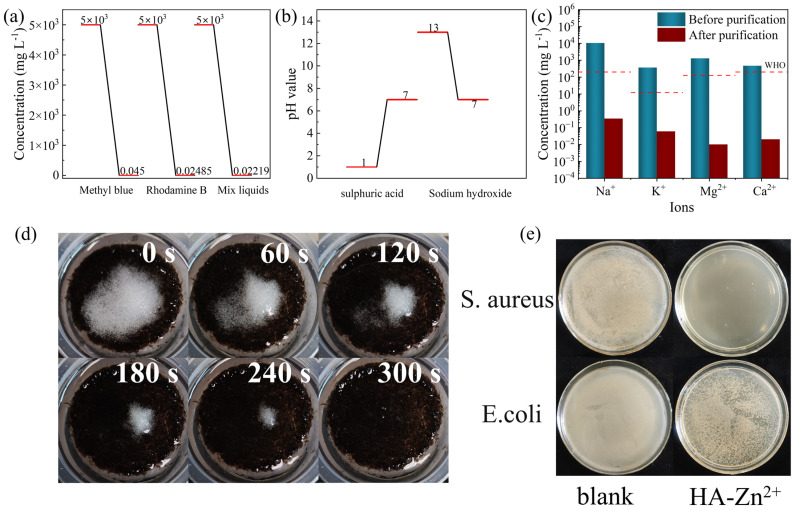
Evaporator water purification. (**a**) Purification of organic dyes. (**b**) Acid–base purification. (**c**) Artificial seawater. (**d**) Salt dissolution image of a PCH. (**e**) Inhibitory effect of PCH on Escherichia coli and Staphylococcus aureus.

In order to investigate the purification ability of the PCH on wastewater, PCH was put into the concentration of 5 g L^−1^ methylene blue solution, rhodamine B solution, and their mixed solution for evaporation treatment, and the evaporated water was collected and detected by ultraviolet-visible spectroscopy to calculate the concentration of the organic dyes. The results are shown in Figure 6a, which shows that the photothermal evaporation treatment has a good purification effect on wastewater polluted with organic dyes. The concentration of methylene blue in the water after photothermal evaporation treatment is only 0.045 mg L^−1^, the concentration of rhodamine B is 0.02485 mg L^−1^, and the concentration of the mixed solution is 0.02219 mg L^−1^, which is much lower than their original concentration. In order to further investigate the purification ability of the PCH, sulfuric acid solution and sodium hydroxide solution with pH values of 1 and 13, respectively, are purified by the PCH and subjected to photothermal evaporation treatment, and the results are shown in Figure 6b; also, the pH value of the solution after the photothermal evaporation treatment is detected to be neutral by pH test strips.

To prove the seawater purification ability of the PCH, using artificial seawater [71] for evaporation test experimental results as shown in Figure 6c. The evaporation of water collected by the content of the four metal ions have dropped by four orders of magnitude, in line with the World Health Organization’s drinking water standards. In addition, we also tested the salt removal ability of the PCH; we placed the PCH in a solution simulating seawater, and placed 1 g NaCl on the surface of the PCH for evaporation experiments. As shown in Figure 6d, NaCl dissolved within 300 s, indicating that the PCH has a good ability to remove salt.

Because Zn^2+^ itself has good antibacterial properties, Zn^2+^ produces significant toxic effects on bacteria by interfering with its carbon metabolism [72]. Co-mixing the PCH with diluted bacterial broth solution in a constant temperature incubator at 37 °C for 24 h, the bacterial mixture is diluted and applied to agar Petri dishes and incubation is continued for 12 h. As shown in Figure 6e, the inhibitory effect of the PCH loaded with Zn^2+^ complex for *S. aureus* is much more than that of the blank control group. Similarly in the experiments with *E. coli*, the PCH loaded with Zn^2+^ complexes also show significant bacterial inhibition. In addition, due to the abundance of CS as well as HA and their low price, PCH has a lower cost than other reported solar water evaporators [73], which is only 1.97 $ m^−2^ (Table S1), so that it has the basis for practical application.

## 3. Conclusions

In summary, in this paper, a novel CS-based aerogel was prepared by combining organic–inorganic materials with biomass materials, cross-linking EP-POSS with CS, and freeze-drying, which has excellent mechanical properties as well as superb water-absorbing capacity. The water absorption rate of PCS can reach 2.67 mm s^−1^ at 30 wt% of EP-POSS, and the water absorption capacity of PCS can reach 22.09 g g^−1^ at 50 wt% of EP-POSS. Moreover, the introduction of hydroxyl groups allows PCS to form hydrogen bonds with H_2_O in water, and PCS has a rapid water-driven self-recovery ability under the effect of hydrogen bonding. Based on the PCS with 30 wt% EP-POSS added, which exhibited the fastest water transport rate, it was utilized as a solar water evaporator by depositing the HA-Zn^2+^ photothermal layer on its top layer. The tested PCH has a stable solar water evaporation capability and due to the underwater self-recovery capability of the PCS, it can remain water-transportable even after external crushing. After 10 consecutive photothermal evaporation tests, the PCH does not show any serious performance degradation, and it has excellent water purification and salt drainage capabilities. In addition, the PCH shows excellent antimicrobial performance due to the antimicrobial properties of Zn^2+^, so that it can meet the needs of water evaporation in harsh environments.

This photothermal aerogel, exhibiting remarkable water-driven recovery capabilities, effectively harnesses its water-driven recovery behavior to adapt to diverse aquatic environments, including those containing numerous rigid objects that could potentially compromise the integrity of the evaporator. Furthermore, its resilience enables its deployment in extremely adverse climatic conditions, mitigating the risk of structural degradation. As such, this evaporator is anticipated to significantly advance the practical utilization of solar energy evaporation systems.

## 4. Materials and Methods

### 4.1. Materials

Chitosan (CS, MW = 30,000, ≥85% deacetylation), ZnCl_2_, and NaBr (analytical purity) were purchased from Shanghai Maclean Biochemical Technology Co. (Shanghai, China). Triethoxy (3-epoxypropoxy) silane (KH-561, purity > 96.0%) and tannin (analytical purity) were purchased from Aladdin Reagent Co. (Shanghai, China). Hydrochloric acid, MgSO_4_, and KCl (analytical purity) were purchased from Yantai Shuang Chemical Co. (Yantai, China). Tetrahydrofuran (THF, analytical purity), methanol (analytical purity), acetic acid (analytical purity), and AlCl_3_ (analytical purity) were purchased from Tianjin Tianli Chemical Reagent Co. (Tianjin, China). MnCl_2_ (analytical purity) was purchased from Tianjin Xinput Chemical Co. (Tianjin, China). NaCl, CaCl_2_, and MgCl_2_ (analytical purity) were purchased from Tianjin Hengxing Chemical Reagent Manufacturing Co. (Tianjin, China). NaHCO_3_ (analytical purity) was purchased from Tianjin Dayong Chemical Reagent Manufacturing Co. (Tianjin, China).

### 4.2. Preparation of PCH

#### 4.2.1. Preparation of the EP-POSS

EP-POSS was directly synthesized by silane hydrolysis; 10 mL of KH-561, 150 mL of methanol, 0.5 mL of concentrated hydrochloric acid, and 0.65 mL of deionized water were placed in a flask and the reaction was carried out with magnetic stirring in an oil bath at 90 °C for 10 days, and the solvent was slowly evaporated by continued magnetic stirring and heating after 10 days until the solution was a pale yellow transparent viscous liquid. Deionized water is added to make the liquid become a white suspension, and the yellowish viscous product EP-POSS can be obtained after freeze-drying.

#### 4.2.2. Preparation of the PCS

An amount of 1.5 g of CS was dissolved in 100 mL of 1% v v^−1^ acetic acid solution, different ratios (0.3 g, 0.9 g, 1.5 g) of EP-POSS were added, and the pH was adjusted to 3 using 1 mol L^−1^ hydrochloric acid, and the reaction was performed with magnetic stirring at room temperature for 48 h. After the reaction was performed with saturated sodium bicarbonate solution, the pH was adjusted to 6.4, and the aqueous gel was obtained by ultrasonication for 5 min to remove air bubbles and water bathing at 50 °C for 2 h. The aqueous gel was frozen and then freeze-dried to obtain PCS with different ratios. Pure CS aerogel was prepared by the same method without adding POSS.

#### 4.2.3. Construction of Photothermal Layers of HA-Zn^2+^ Complexes

A total of 4 g of tannin was dissolved in 100 mL water, hydrothermal reaction was carried out at 240 °C for 4 h, the reaction solution was filtered by 0.45 μm filter membrane, and the filtered solution was dried to obtain HA. After mixing an aqueous HA solution of 1 g L^−1^ with Zn^2+^ solutions of different concentrations (0.5 mmol L^−1^, 1 mmol L^−1^, 5 mmol L^−1^, and 10 mmol L^−1^) 1:1, the mixed solution was added to the above PCS solution, and the pH was adjusted to 6.4 by sodium bicarbonate, which was then covered on the above aqueous gel, freeze it as a whole for 24 h, and then freeze-dried to obtain PCH. The evaporator containing tannin metal complexes was prepared according to the above method, and the tannin concentration is 1 g L^−1^.

### 4.3. Structure Characterizations

Fourier Transform Infrared spectrometer (FTIR, PerkinElmer Frontier spectrometer, PerkinElmer, Waltham, MA, USA, measuring range 4000–500, resolution 2 cm^−1^), UV-visible spectrophotometer (TU-1901, Beijing Pulse Analytical, Beijing, China), X-ray photoelectron spectrometer (XPS, Thermo Kalpha, ThermoFisher Technologies, Waltham, MA, USA, using K-Alpha X-rays), and Fourier Transform Ion Cyclotron mass spectrometry (FT-ICR-MS, MALDI-SolariX FTMS, Bruker, Billerica, MA, USA, with samples dissolved in a solvent of toluene: methanol 9:1) were used for the analysis of the chemical structure and composition of the EP-POSS and PCS. Scanning electron microscope (SEM, TM3030, Hitachi, Tokyo, Japan, sample gold spraying treatment, acceleration voltage 5 kV, working distance 2–40 mm) was used for the micro-morphological analysis of the aerogels. Universal mechanical testing machine (UTM-2203, Shenzhen Sansi Zongheng Technology Co., Ltd., Shenzhen, China, compression rate 5 mm min^−1^, room temperature, vertically compressed) was used to test the mechanical properties of the aerogels, and the compression modulus was determined by the slope of the stress–strain curve at low strain. A video optical contact angle meter (OCA20, Data-physics, Baden-Wuerttemberg, Filderstadt, Germany, 5 μL water droplets) was used to measure the aqueous contact angle of the aerogels. A Thermogravimetric analyzer was also used (TGAS, STA 6000-SQ8 PerkinElmer, Waltham, MA, USA, measuring range 40–800 °C, heating rate 10 °C min^−1^, nitrogen protection flow rate of 20 mL min^−1^).

### 4.4. Water Absorption Test

The water absorption capacity (C_0_ g g^−1^) was measured by placing the PCS with a mass of m_0_ in water. The mass m_x_ was measured by removing the surface liquid from the PCS after placing it in water for 30 s. The formula is shown below:

$$C_{0}=\frac{m_{x}-m_{0}}{m_{0}}$$

### 4.5. Evaporation Performance Test

The solar evaporation experiments were performed at an indoor temperature of 25 ± 1 °C using a xenon lamp light source (CEL-HXF300, Beijing CEC Jinyuan, Beijing, China) as the sunlight simulator and a solar power meter (SM206- SOLAR, Shenzhen Xinbao Science Instrument, Shenzhen, China) was used to determine the average light intensity on the evaporated surface as 1 kW m^−2^. The PCH was placed in a beaker filled with water, and to avoid the influence of the heat of the light source it was placed 40 cm below the light source, and the mass change was recorded by an electronic balance (FA2104, Shanghai Shunyu Hengping, Shanghai, China). An infrared thermal imager (Uti260A, Haikangweishi, Hangzhou, Zhejiang, China) was used to take infrared pictures and measure the surface temperature. The photothermal purification experiments were carried out by a double-beam UV-visible spectrophotometer to determine the concentration of the organic dyes and determine their pH by a pH meter (PB-10, Sartorius, Gottingen, Lower Saxony, Germany).

### 4.6. Artificial Seawater Purification Test

Artificial seawater was formulated with the following composition: 26.5 g NaCl, 3.3 g MgSO_4_, 2.4 g MgCl_2_, 1.3 g CaCl_2_, 0.7 g KCl, 0.2 g NaHCO_3_, and 0.08 g NaBr dissolved in 1 L of water. The evaporated water was collected and tested for metal ions using an ICP-OES (Agilent 5800 ICP-OES, Santa Clara, CA, USA).

### 4.7. Antimicrobial Performance Test

Bacteria were scraped into sterile tubes and saline-fixed to 1.5 × 10^6^ CFU mL^−1^ before bacterial solution was added. After diluting the bacterial solution 50-fold using beef broth, 1 mL of the mixed solution was taken and added to 9 mL of the saline and stored at 37 °C for 24 h. The mixed solution was diluted 10-fold using saline, and 20 μL was taken and spread evenly in the agar medium and incubated at 37 °C for 12 h.

## Data Availability

Data are contained within the article.

## Supplementary Materials

The following supporting information can be downloaded at: https://www.mdpi.com/article/10.3390/gels10040279/s1, Figure S1. EP-POSS structure inferred from FT-ICR-MS and FT-IR spectra (a) Located at 578 m/z and (b)750 m/z; Figure S2. XPS total spectra of aerogels, (a) pure CS aerogel and (b) PCS aerogel; Figure S3. XRD pattern of CS and PCS; Figure S4. TG and DTG curves of the photothermal layer; Figure S5. N_2_ adsorption–desorption isothermal curves of pure chitosan aerogel and PCS; Figure S6. Water contact angle of pure chitosan aerogels; Figure S7. Before and after comparison after ultrasound simulation; Figure S8. Photothermal evaporation experimental setup diagram; Figure S9. Infrared thermogram of dry sample under a strong sunlight, (a) photothermal layer with added tannin-Zn^2+^, (b) PCS, and (c) photothermal layer with added HA; Table S1. PCH solar water evaporator material cost analysis; Video S1 showing PCS–water contact angle (mp4); Video S2 showing pure CS aerogel–water contact angle (mp4); Video S3 showing PCS water-driven recovery (mp4); Video S4 showing pure CS aerogel immersed in water (mp4); Video S5 showing pure CS aerogel water-driven recovery (mp4); Video S6 showing PCS immersion in water (mp4); Video S7 showing simulating an outdoor environment with an ultrasonic cleaner (mp4).
