# Supplementary material for: Polyhedral Oligomeric Sesquioxane Cross-Linked Chitosan-Based Multi-Effective Aerogel Preparation and Its Water-Driven Recovery Mechanism
Source: Gels. 2024 Apr 20;10(4):279. doi: 10.3390/gels10040279 (PMC11049377; doi:10.3390/gels10040279)
Supplement: Supplementary file 1 [file gels-10-00279-s001.zip › gels-2953364-supplementary.pdf]

Supporting Information

POSS cross-linked chitosan-based multi-effective aerogel preparation and its water-driven recovery mechanism

Section 1：Morphological and structural characterisation of EP-POSS and PCH

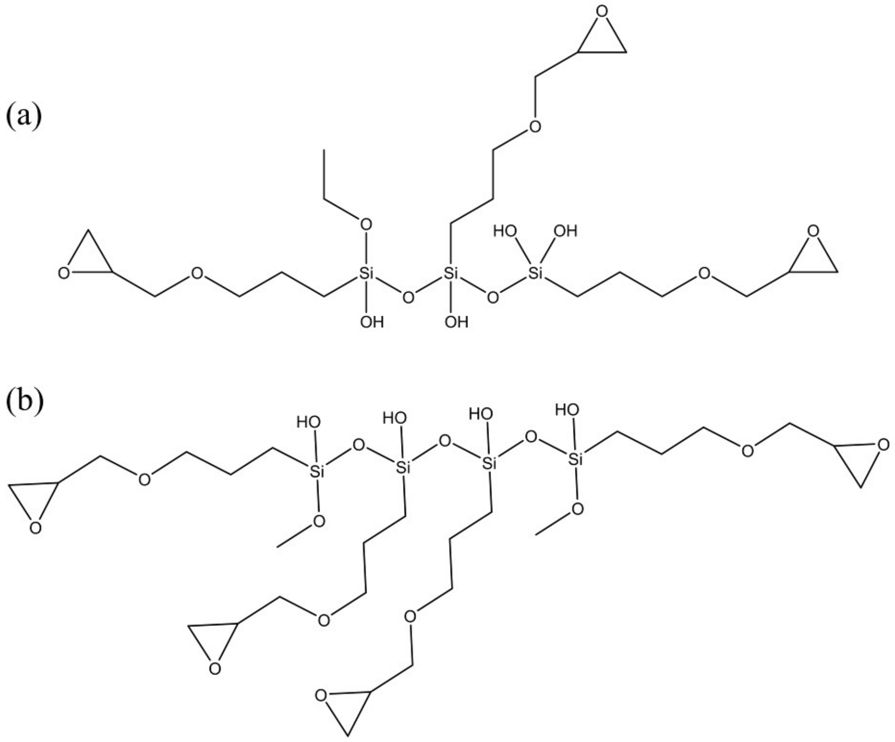

Figure S1. EP-POSS structure inferred from FT-ICR-MS and FT-IR spectra (a) Located at 578 m/z, (b)750 m/z

XPS testing: characterisation of the chemical structure of the sample using K-Alpha x-ray photoelectron spectroscopy

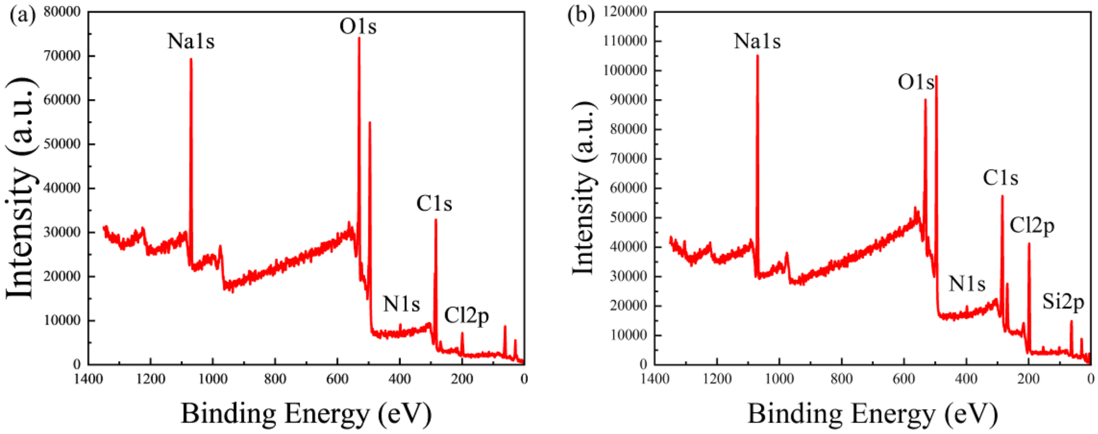

Figure S2. XPS total spectra of aerogels, (a) pure CS aerogel, (b) PCS aerogel

XRD tests: X-ray diffraction patterns were carried out using a CuKa (λ = 1.542 Å) radiation monochromator with a scanning range of 10-90°.

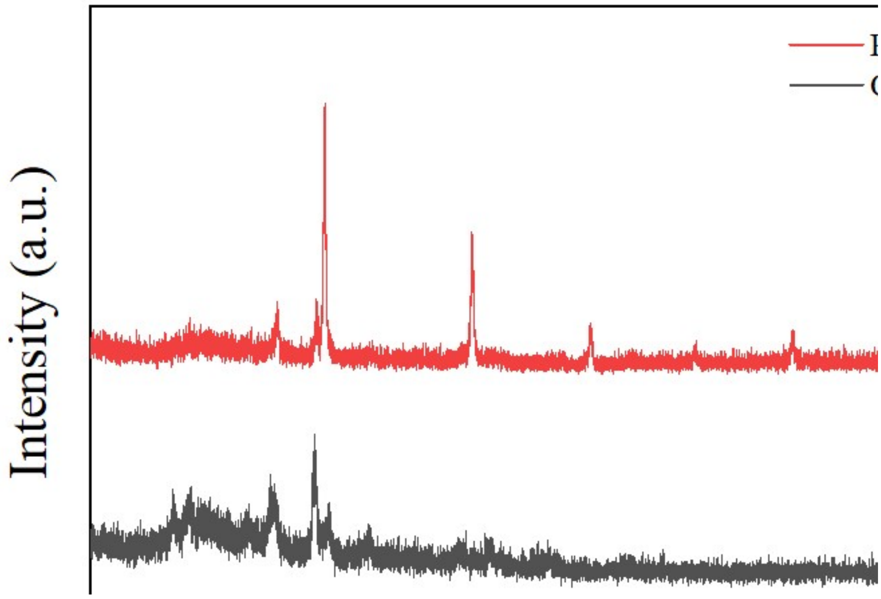

Figure S3. XRD pattern of CS, PCS

From XRD, it can be seen that pure CS aerogels and PCS only have crystalline peaks of NaCl.

Thermal stability test: Test analysis using the STA6000-SQ8 analyser, ramping from room temperature to 800°C at a rate of 10°C/min under nitrogen protection.

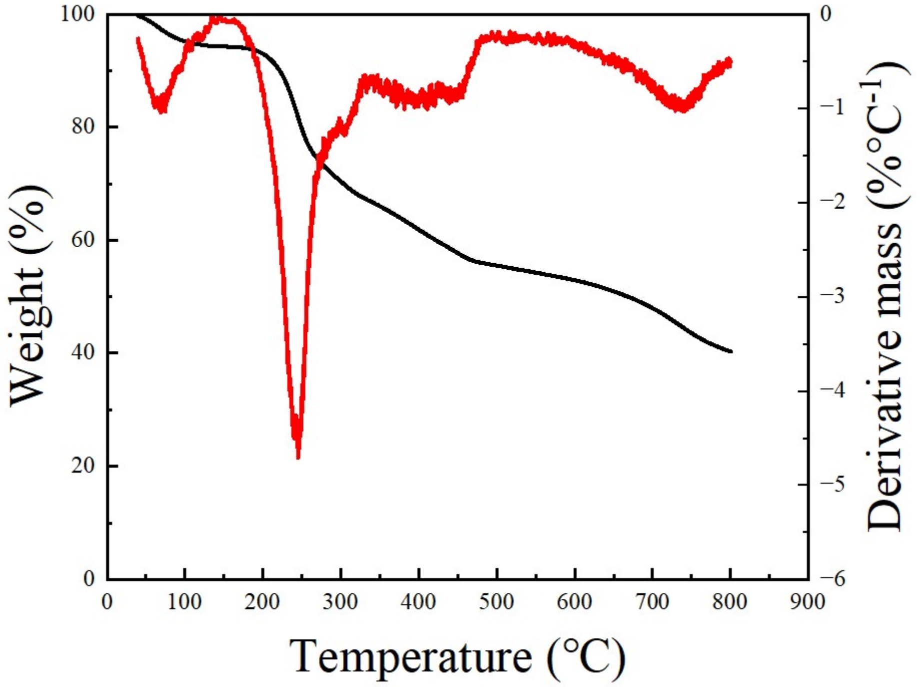

Figure S4. TG, DTG curves of the photothermal layer

From the curves, it can be seen that the thermal decomposition of the photothermal layer is similarly divided into three stages, with the evaporation of free and bound water in the first stage, the decomposition of the CS chain in the second stage, and the continuation of charring in the third stage.

BET tests: Sample testing with the ASAP2460 pore size analyzer

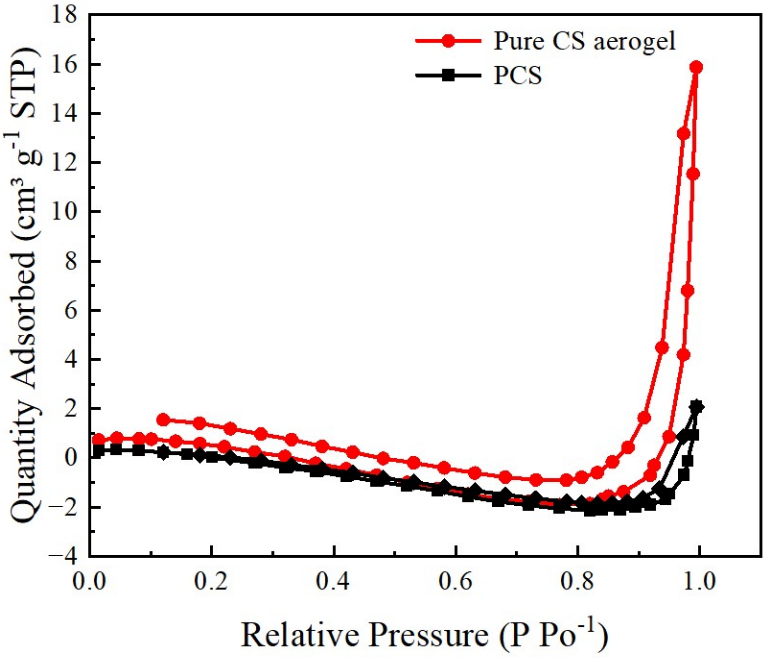

Figure S5. N_2 adsorption-desorption isothermal curves of pure chitosan aerogel and PCS

The test results indicate that the average pore size for adsorption of pure CS aerogel is 138.0719 nm, and its specific surface area is 0.7115 m²/g. However, after the addition of EP-POSS, the average pore size for adsorption of PCS decreases to 54.4524 nm, and its specific surface area is 0.2360 m²/g.

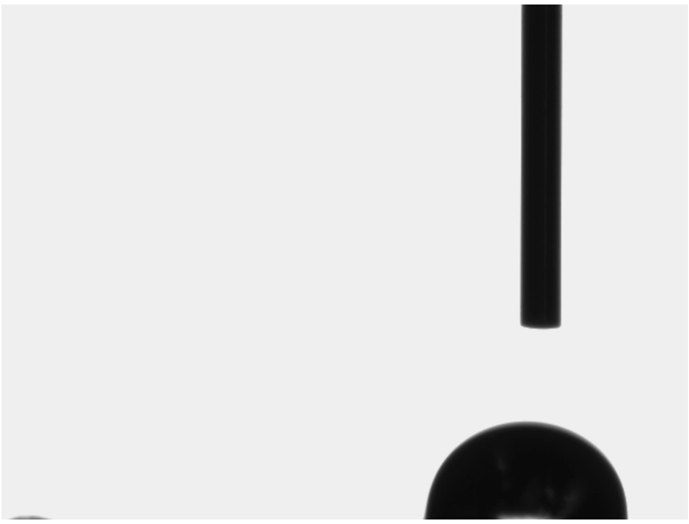

Figure S6. Water contact angle of pure chitosan aerogels.

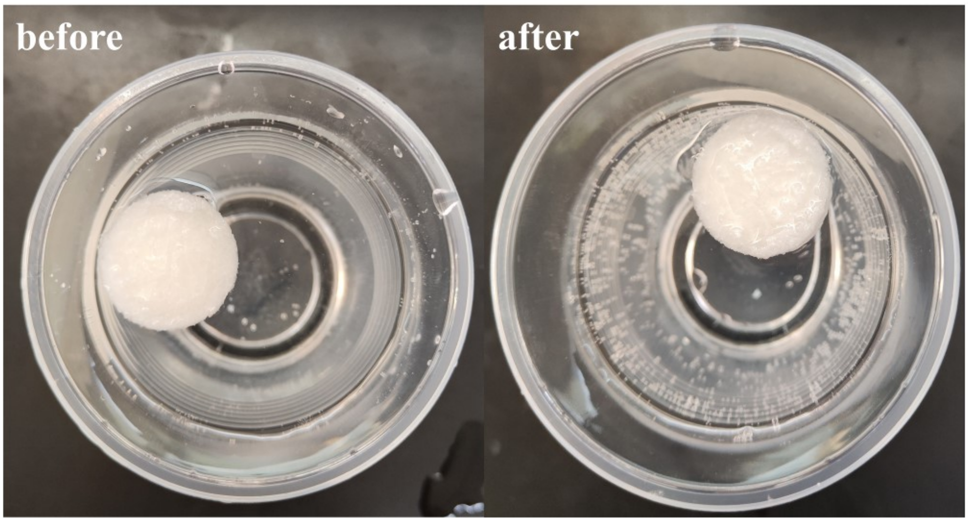

Figure S7. Before and after comparison after ultrasound simulation

Section 2：Photothermal performance of PCH and solar water evaporation performance test

Photo-Hot Water Evaporation Test: A CEL-HXF300 full-band xenon lamp is used to simulate sunlight, and the amount of water lost is recorded at one solar light intensity, with the test sample 40cm away from the light source to avoid the effects of heat from the light source.

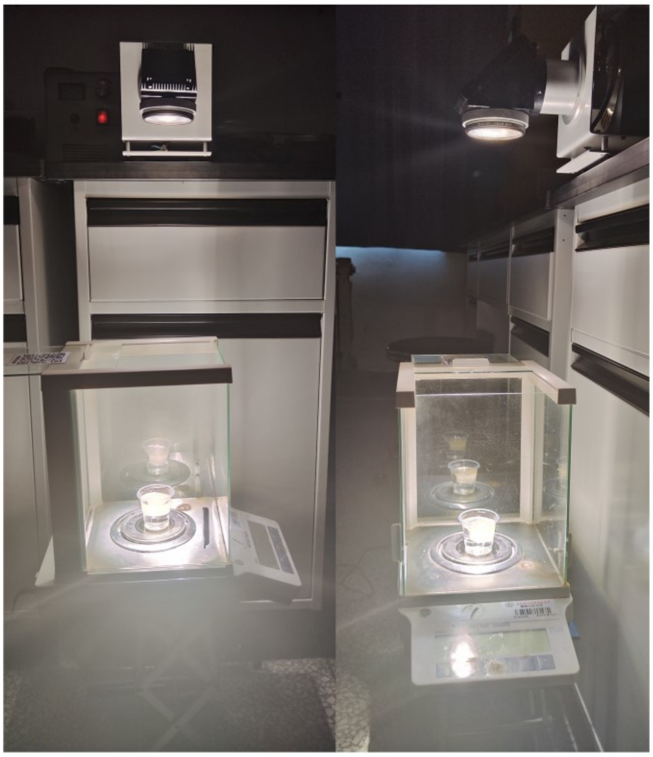

Figure S8. Photothermal evaporation experimental setup diagram

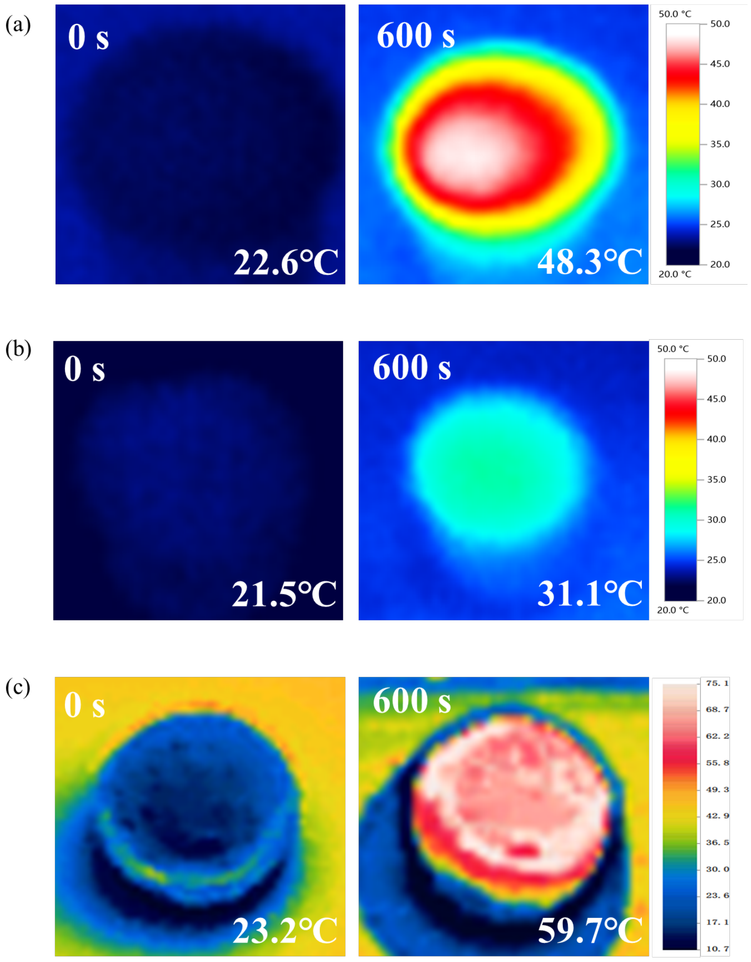

Figure S9. Infrared thermogram of dry sample under a strong sunlight, (a) photothermal layer with added tannin-Zn^{2+}, (b) PCS, (c) photothermal layer with added HA

Evaporator material cost analysis: The PCH solar evaporator in this paper has a thickness of 1 cm and a density of 11.33 mg/cm^3; therefore, the mass of 1 m^2 evaporator is 0.1133 kg. **Table S1** lists the mass and cost of a 1 m^2 evaporator.

Table S1 PCH solar water evaporator material cost analysis

materials	Mass (kg/m^2)	Unit price ($/kg)	Cost ($/m^2)
Chitosan	0.0708	20	1.416
KH-561	0.0706	6.8	0.48008
Tannin	0.016	4.8	0.0768
$ZnCl_2$	0.0011	1.3	0.00143
Total material cost			1.97431

From Table S1, it is shown that the raw material cost of the PCH solar water evaporator is ~1.97$/m^2.
